# Emollient satisfaction questionnaire: validation study in children with eczema

**DOI:** 10.1111/ced.15189

**Published:** 2022-05-16

**Authors:** Georgia G. Rowley, Stephanie J. MacNeill, Matthew J. Ridd

**Affiliations:** ^1^ Population Health Sciences Bristol Medical School Bristol UK; ^2^ Bristol Trials Centre Bristol Medical School University of Bristol Bristol UK

## Abstract

**Background:**

Emollients are used as maintenance therapy for all severities of eczema but there is a lack of head‐to‐head comparisons of effectiveness and acceptability.

**Aim:**

To determine the validity of a self‐report questionnaire designed to assess user satisfaction with a given emollient and to report the findings.

**Methods:**

Data were analysed from the Choice of Moisturiser for Eczema Treatment trial, which compared four emollient types (Aveeno^®^ lotion, Diprobase^®^ cream, Doublebase^®^ gel and Hydromol^®^ ointment) in children aged < 5 years with clinically diagnosed eczema. An emollient satisfaction questionnaire was completed after 12 weeks. Responses for individual items were scored from 0 to 4. Total scores ranged from 0 to 28 (low to high satisfaction). Completion rates and distributions of responses for individual items and total scores, categorized by emollient type, were assessed, and two hypotheses were tested to determine the questionnaire's construct validity.

**Results:**

Data from 77.2% (152 of 197) of participants were analysed. One item was rejected because of a high rate (44.7%) of ‘don't know’ responses, leaving seven items with high completion rates (98.7%) and weak evidence of floor or ceiling effects. A positive association was observed between total score and overall emollient satisfaction (Spearman correlation 0.78; *P* < 0.001). Total scores were highest (mean ± SD 23.5 ± 3.9) in the lotion group and lowest (18.4 ± 4.6) in the ointment group.

**Conclusion:**

The emollient satisfaction questionnaire appears to have good validity. Further work is required to validate the questionnaire in other settings and to assess its reliability.

## Graphical abstract

Despite emollient use being the maintenance therapy for all severities of eczema, there is a lack of head‐to‐head comparisons of effectiveness and acceptability. We sought to assess the validity of a self‐report questionnaire, which assesses emollient satisfaction. The questionnaire appears to have good validity but further work is required to assess reliability and to validate its use in other settings.

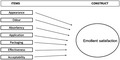

## Introduction

Eczema (also termed atopic eczema or atopic dermatitis) is a chronic inflammatory skin condition predominately affecting children.^1^, ^2^ Treatment guidelines recommend a stepped approach to management, tailoring treatment to disease severity.^3^ Leave‐on emollients are recommended as maintenance therapy for all severities of the disease. There is a wide variety of emollients available, which vary in consistency from watery lotions, through creams and gels, to solid ointments.^4^ The most common reason for treatment failure is underuse of topical therapies.^5^

There is reasonable evidence that emollients improve disease severity, and may reduce the need for corticosteroids, but there are limited data on the relative effectiveness (and user acceptability) of different types. A Cochrane review identified 77 trials evaluating the effectiveness of emollients,^1^ but the authors were unable to conclude whether some of the emollients, or their ingredients, are better than others. Head‐to‐head comparisons in clinical randomized controlled trials can be challenging,^6^, ^7^ and are expensive to undertake. While satisfaction questionnaires can be included as outcomes in trials, they can also be used in cross‐sectional studies as an economical means of obtaining opinions.^8^ However, the few published ‘emollient satisfaction’ questionnaires lack evidence of their validity, i.e. ‘the degree to which an instrument truly measures the construct(s) it purports to measure’.^9^ Therefore, we aimed to assess the validity of a questionnaire designed to assess user satisfaction with a given emollient, the Emollient Satisfaction Questionnaire (ESQ), which was used in the Choice of Moisturiser for Eczema Treatment (COMET) trial.^10^

## Methods

### The Choice of Moisturiser for Eczema Treatment trial

Data for this study were drawn from the COMET feasibility trial,^10^ which was a study to determine the feasibility of a clinical trial comparing four different leave‐on emollients [Aveeno^®^ lotion (Johnson & Johnson, Brunswick, NJ, USA), Diprobase^®^ cream (Bayer UK, Reading, Berkshire, UK), Doublebase^®^ gel (Dermal Laboratories, Hitchin, Hertfordshire, UK) and Hydromol^®^ ointment (Alliance Pharmaceuticals, Chippenham, Wiltshire, UK)] for the treatment of 197 children, aged 1 month to < 5 years, with a clinical diagnosis of eczema. Participants were recruited via general practices in the west of England and followed up for 12 weeks. The COMET trial was approved by the Central Bristol Research Ethics Committee (no. 13/SW/0297) and clinical trial authorization given by the Medicines and Healthcare products Regulatory Agency (MHRA) (no. 03299/0017/001–003).^11^

### The Emollient Satisfaction Questionnaire

User satisfaction with an emollient cannot be directly observed; the ESQ was developed by one of the coauthors (MJR) of this study, after reviewing existing literature at the time. Emollient satisfaction was defined as ‘the extent to which parents positively rated their given emollient for specific factors, such as absorbency’. Although this questionnaire was completed by parents/carers at 12 weeks as part of this clinical trial (COMET), it was intended that the questionnaire could be used in other settings, for example in clinics.

**Figure 1:**
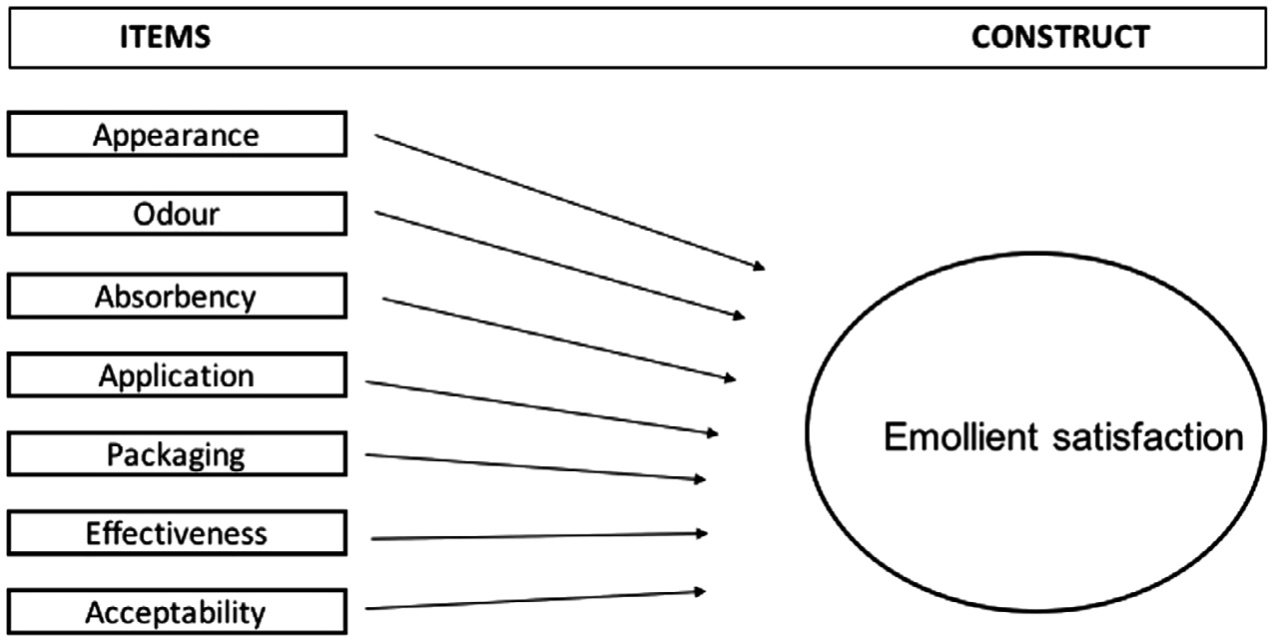
Formative model reflecting the relationship between items and the construct, specific to the ESQ.

Conceptually, multi‐item instruments can be reflective or formative, which are models that represent the relationship between the items and the construct to be measured.^9^ Applying the modified Jarvis *et al*. checklist (Supplementary Table S1),^11^ we determined that the formative model was the most appropriate model for this study. Each item of the ESQ referred to a unique concept and contributed a part of the construct; when combined together, the total items formed the whole construct (parental emollient satisfaction)^9^ (Fig. 1). COSMIN (COnsensus‐based Standards for the selection of health Measurement Instruments) guidelines state that evaluating the internal structure of a measure, using the measurement properties of structural validity, internal consistency and crosscultural validity/measurement invariance, is only relevant for reflective models (Supplementary Data S1).^12^ Therefore, these measurement properties were not evaluated in this study.

**Figure 2:**
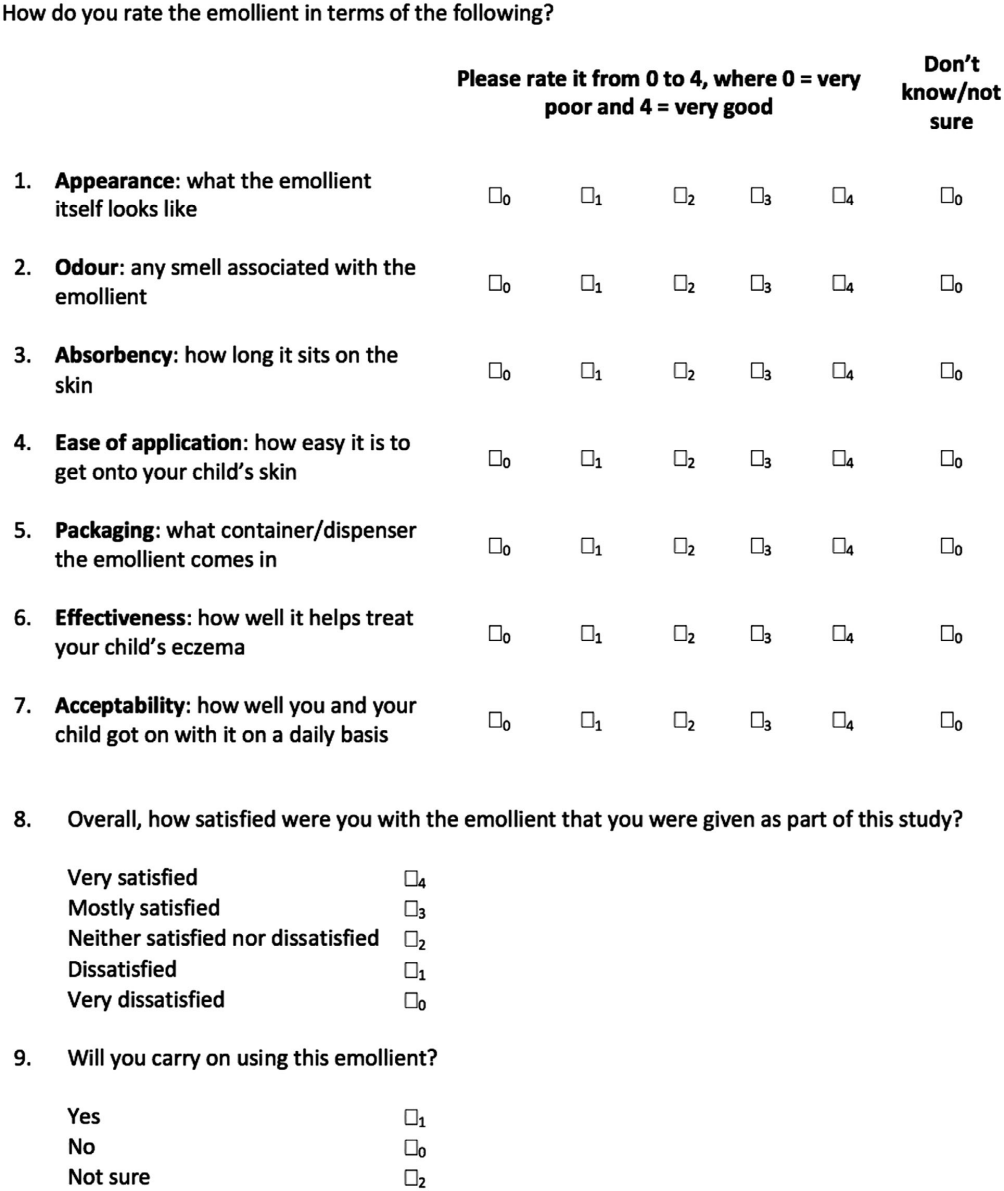
Analysed version of the Emollient Satisfaction Questionnaire (ESQ) from the Choice of Moisturiser for Eczema Treatment (COMET) trial. This is the version of the questionnaire we recommend be used in future studies.

The ESQ asked participants to score their allocated emollient on appearance, odour, absorbency, application, packaging, effectiveness and acceptability, using five response categories (from ‘very poor’ to ‘very good’) plus a ‘don't know’ option. We chose to omit an eighth item from the originally administered questionnaire (‘Ingredients: what's in it/what it is made from’) due to a large proportion (*n* = 68, 44.7%) of ‘don't know’ responses, compared with a low proportion ≤ 3.6%) of ‘don't know’ responses to other items. Two further questions assessed overall emollient satisfaction and intention for continued use of that emollient, with a space for optional free text. The analysed version of the ESQ is presented in Fig. 2.

A total scaled emollient satisfaction score was calculated by summing the individual scores (0–4) of Questions 1–7. We used unweighted scores for each item as we deemed each item to equally represent the construct of interest (emollient satisfaction). Only participants who had completed all seven questions without any ‘don't know’ responses were included in total score calculations. Therefore, total scaled emollient satisfaction scores ranged from 0 to 28 (low to high satisfaction).

### Hypotheses formulation

To assess the construct validity of the ESQ, we formulated and tested the following two hypotheses.

#### Hypothesis 1

Emollient satisfaction is higher for participants who give a higher overall rating for their allocated emollient. We assessed this by comparing total scaled emollient satisfaction score with overall emollient satisfaction item: ‘Overall, how satisfied were you with the emollient that you were given as part of this study?’.

#### Hypothesis 2

Emollient satisfaction is higher for participants who intend to continue using their allocated emollient; we assessed this by comparing total scaled emollient satisfaction score and overall emollient satisfaction item with intention for continued emollient use item: ‘Will you carry on using this emollient?’.

Free‐text comments were sought to support or refute the validity of individual items or of the questionnaire overall.

### Data analysis

Data were imported into using STATA software (V16.0; StataCorp, College Station, TX, USA) and all analyses were performed using this software. Descriptive statistics were used to assess the baseline characteristics of participants, and the distributions of individual and total scaled satisfaction scores (both overall distribution and by emollient type). Mean ± SD or median and interquartile range (IQR) were used for continuous data. Frequencies and percentages were used for categorical data. Completion rates of individual questionnaire items were assessed. Each item was assessed for floor and ceiling effects by calculating the proportion of participants selecting the lowest and highest response options, respectively, for each item. We did not prespecify what constituted a floor/ceiling effect, but conventionally this can be between 5% and 15% of responses.^13^ The χ^2^ test, Spearman rank correlation coefficient and Kruskal–Wallis tests with Bonferroni correction were performed as appropriate to explore associations.

## Results

### Participant demographics

**Figure 3:**
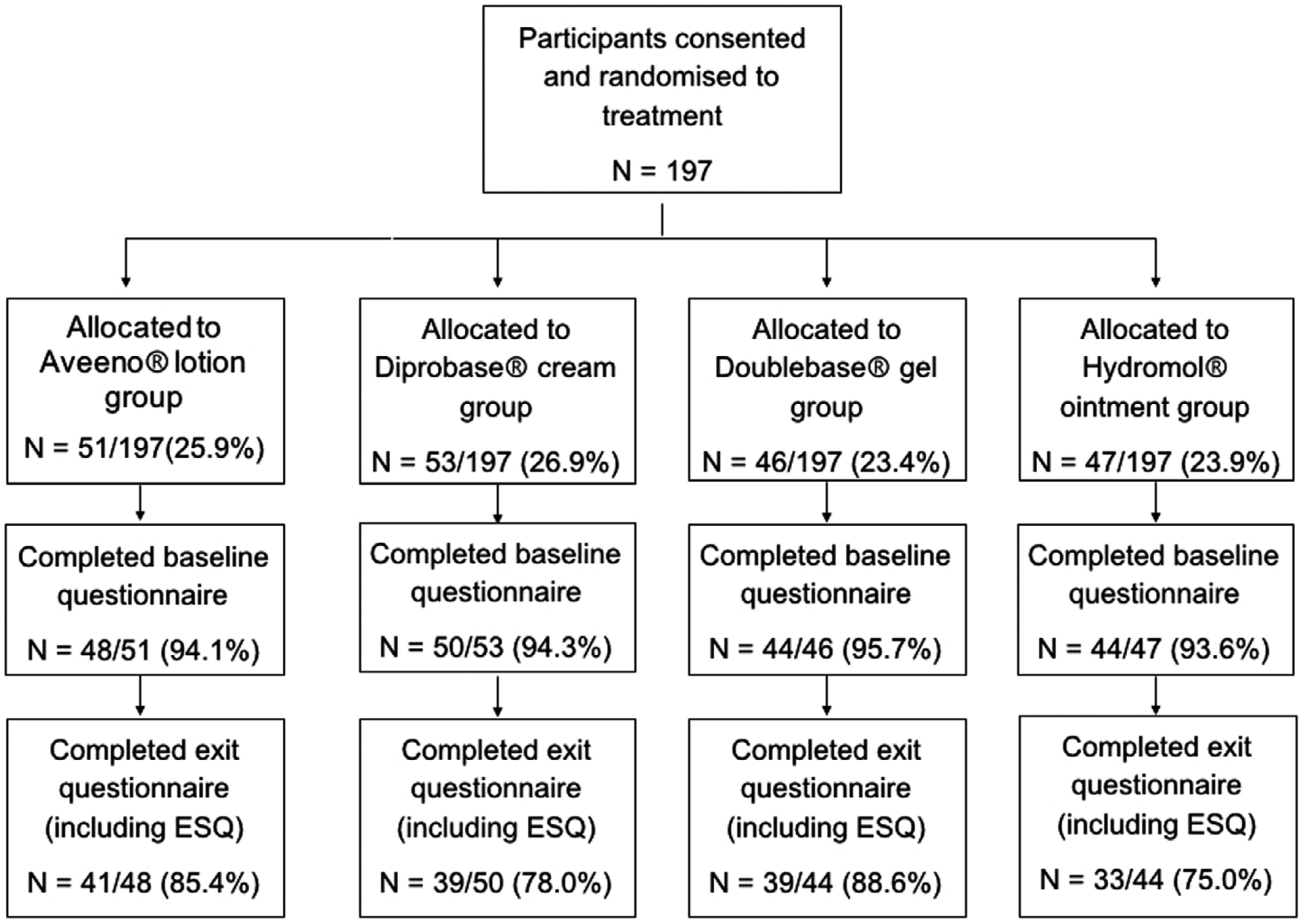
Flow of participants throughout the Choice of Moisturiser for Eczema Treatment (COMET) trial, from randomization of participants to completion of the Emollient Satisfaction Questionnaire (ESQ).

In total, 197 participants were recruited into the COMET trial, of whom 152 (77.2%) completed the baseline questionnaire and the ESQ at Week 12. A participant flow diagram is presented in Fig. 3. Of the participants with completed ESQ (*n* = 152), the mean age at baseline was 1.4 ± 1.0 years. Most were white (89.3%) with mild to moderate eczema (mean baseline POEM score 8.3 ± 5.5) (Table 1). Participants who did not have a completed ESQ (*n* = 45) tended to be younger (mean 1.0 ± 1.1 years; *P* < 0.03), and were less likely to be white (66.7%, *P* = 0.001) (Table 1).

**Table 1:** Baseline demographics of participants.^a^

Parameter	Participants			*P* (test)^b^
	Randomized (*n* = 197)	Complete ESQ (*n* = 152)	Missing ESQ (*n* = 45)	
Sex, *n* (%)				
Male	112 (56.9)	91 (59.9)	21 (46.7)	0.12 (χ^2^)
Female	85 (43.1)	61 (40.1)	24 (53.3)	
Ethnicity, *n* (%)				
White	155 (85.2)	133 (89.3)	22 (66.7)	0.001 (χ^2^)
Non‐white	27 (14.8)	16 (10.7)	11 (33.3)	
Data missing	15	3	12	
Age, years				
Mean ± SD	1.3 ± 1.1	1.4 ± 1.0	1.0 ± 1.1	0.03 (*t*‐test)
Median (IQR)	1.0 (0–2)	1.0 (1–2)	1.0 (0–1)	
Emollient allocation, *n* (%)				
Aveeno lotion	51 (25.9)	41 (27.0)	10 (22.2)	0.32 (χ^2^)
Hydromol ointment	47 (23.9)	33 (21.7)	14 (31.1)	
Diprobase cream	53 (26.9)	39 (25.7)	14 (31.1)	
Doublebase gel	46 (23.4)	39 (25.7)	7 (15.6)	
Baseline POEM severity score				
Mean ± SD	8.8 ± 5.9	8.3 ± 5.5	10.5 ± 6.9	0.02 (*t*‐test)
Median (IQR)	8.0 (4–12)	8.0 (4–12)	9.5 (5–14.75)	
Eczema category, *n* (%)				
Clear or almost clear	27 (13.8)	24 (15.8)	3 (6.8)	0.02 (χ^2^)
Mild	65 (33.2)	50 (32.9)	15 (34.1)	
Moderate	83 (42.3)	67 (44.1)	16 (36.4)	
Severe/very severe	21 (10.7)	11 (7.2)	10 (22.7)	
Data missing	1	0	1	
UKWP eczema diagnostic criteria, *n* (%)				
Met the criteria	–	89 (58.6)	22 (64.7)	0.51 (χ^2^)
Did not meet the criteria	–	63 (41.4)	12 (35.3)	
Data missing	–	0	11	

ESQ, Emollient Satisfaction Questionnaire; POEM, Patient Oriented Eczema Measure; UKWP, United Kingdom Working Party.

^a^ Participants are stratified by randomized participants, participants with a completed ESQ and participants with a missing ESQ.

^b^ Statistical comparison refers to the 152 vs. 45 participants with a complete and missing ESQ, respectively.

### Completion and distribution of scaled emollient satisfaction items

Individual ESQ items (Questions 1–7) had high completion rates (i.e. participants gave either a score of 0–4 or a ‘don't know’ response) of 98.7%, although responses were positively skewed (Supplementary Fig. S1). Question 4 (‘application’, 52%) and Question 6 (‘effectiveness’, 9.2%) had the greatest proportion of participants selecting the highest and lowest response categories, respectively. There was weak evidence of a floor effect, although a possible ceiling effect could not be excluded. The mean ± SD and median (IQR) scores of individual ESQ items are presented in Supplementary Table S2.

**Figure 4:**
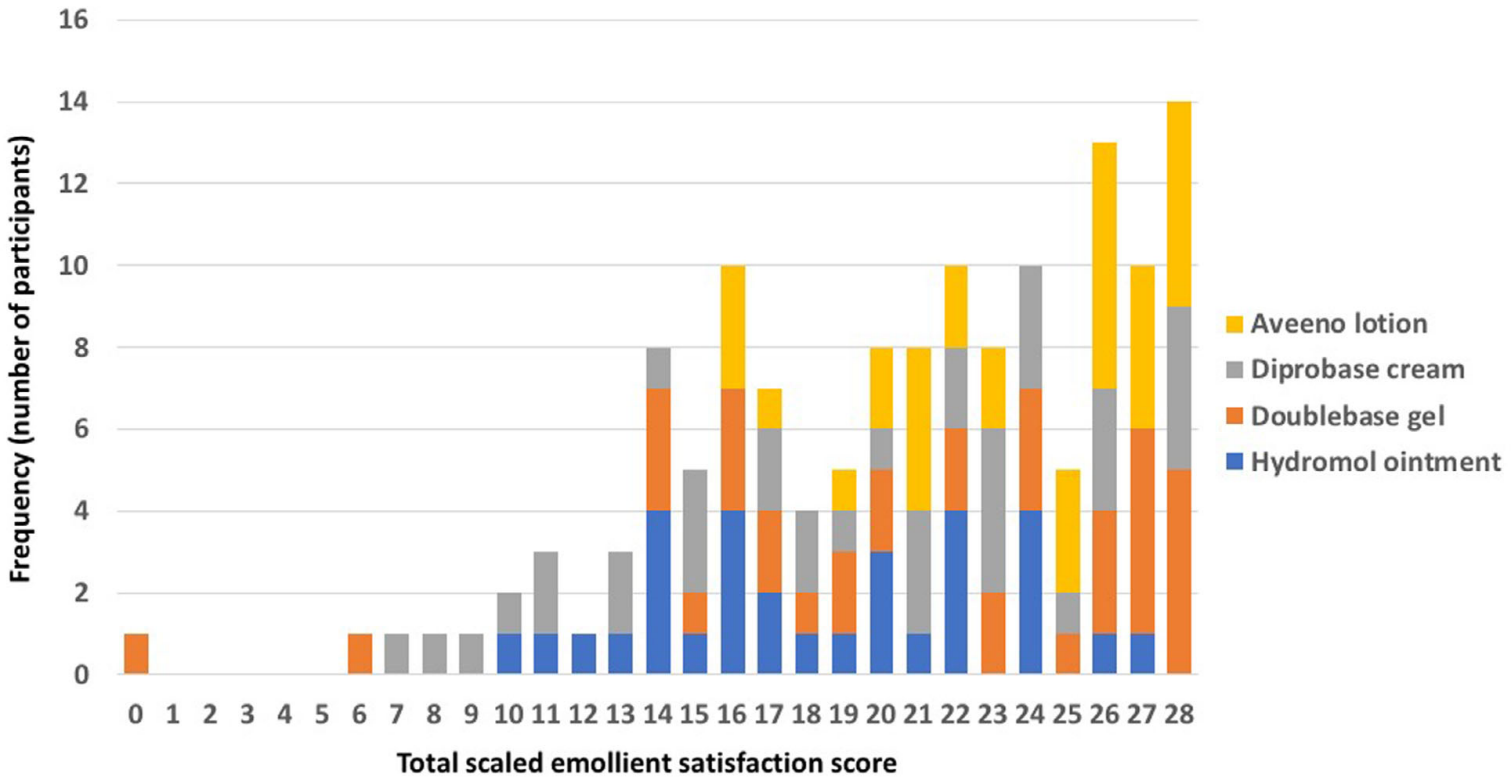
Distribution of responses for total scaled emollient satisfaction score, by emollient type (*n* = 139). [Colour figure can be viewed at wileyonlinelibrary.com]

Of the 152 participants, 139 (91.4%) completed all seven items (either giving a score of 0–4 or a ‘don't know’ response). The full range of scaled scores were observed (from 0 to 28, low to high satisfaction) with a mean ± SD score of 20.6 ± 5.7 and a median (IQR) score of 22.0 (16–26). There was a reasonable distribution of total scaled emollient satisfaction scores, varying by emollient type (Fig. 4).

As shown in Supplementary Table S3, Aveeno lotion had the highest mean total scaled satisfaction score (23.5 ± 3.9), whereas Hydromol ointment had the lowest (18.4 ± 4.6). There was strong evidence of a difference between allocated study emollient and total scaled emollient satisfaction scores (Kruskal–Wallis, *P* = 0.001). A pairwise comparison of means (with Bonferroni correction) demonstrated that there was a difference in total scaled emollient satisfaction scores between Hydromol ointment and Aveeno lotion (*P* = 0.001); and between Diprobase cream and Aveeno lotion (*P* < 0.03).

### Completion and distribution of overall satisfaction and future intention items

The overall emollient satisfaction score item (*n* = 151) was mean ± SD 2.8 ± 1.2 and median (IQR) 3.0 (2–4) (Supplementary Table S4). There was no evidence of any difference between ‘yes’ responses for continued emollient use between the four emollient groups (*n* = 150, χ^2^, *P* < 0.26) (Supplementary Table S5).

### Construct validity

#### Hypothesis 1

The single overall satisfaction item positively correlated with the mean total scaled emollient satisfaction score (Spearman rank correlation coefficient = 0.78, *P* < 0.001) (Supplementary Fig. S2).

#### Hypothesis 2

Intention for continued emollient use was associated with mean total scaled emollient satisfaction scores (Kruskal–Wallis, *P* < 0.001), with the highest scores for those who intended to continue emollient use (Table 2).

**Table 2:** Responses to Question 9^a^ and corresponding satisfaction scores.

Intention to continue use^a^	*n*	Total score, mean ± SD^b^
Yes	87	23.4 ± 4.1
No	27	14.1 ± 4.9
Not sure	25	18.0 ± 4.5
Data missing	13	–
Total	152	–

^a^ Question 9 referred to intention to continue use of that emollient.

^b^ Total scaled emollient satisfaction score.

### Free‐text comments

Of the 152 participants, 118 (77.6%) made free‐text comments. The free‐text comments were consistent with, and provided a rationale for, the individual item scores. There were no comments criticizing the items or the questionnaire more generally.

## Discussion

Our findings represent an initial validation of the ESQ. We omitted one question (regarding ingredients) of the original questionnaire due to a high proportion of ‘don't know’ (*n* = 68; 44.7%) responses. However, the remaining seven items had high (98.7%) completion rates. There was weak evidence of any floor effect; and although we could not exclude a possible ceiling effect, there was a reasonable distribution in total scaled emollient satisfaction scores, which varied by emollient type (Fig. 4). The expected relationships between ESQ and both overall satisfaction with and intention to continue using a given emollient were observed.

The responses for individual items and total scaled emollient satisfaction scores for all emollient groups were positively skewed: as overall emollient satisfaction scores increased, so did the total scaled emollient satisfaction scores. Those who expressed a desire to continue using their emollient also had higher scaled emollient satisfaction scores. Total scaled emollient satisfaction was highest in the Aveeno lotion group and lowest in the Hydromol ointment group.

This is the first study, and the largest of its kind, to evaluate the performance of an emollient satisfaction questionnaire for four different emollient types. Although our data were from an unpowered feasibility trial, we still observed differences in total scaled emollient satisfaction scores and overall satisfaction scores between allocated emollient groups. However, statistical evidence does not necessarily translate into clinical significance. Thus, more work is required to identify a clinically important difference in the ESQ.

Question items were determined by a narrative review of the literature only, and the ESQ was not piloted before being used in the COMET trial. Initial development of the questionnaire would have benefited from qualitative work with patients to determine the relevance, comprehensiveness and comprehensibility of items. However, the free‐text comments we elicited in the current study did not suggest that important factors were missed or that any questions were misunderstood, which was also supported by our quantitative findings (with the exception of an eighth question, which we removed). The ESQ is not worded explicitly in terms of satisfaction ‘overall’ or with respect to specific situations or body sites, which are factors that may change respondent's answers. For formative constructs, there is no way of knowing whether the intended or described construct is complete. The only criterion is that the measurement model matches the definition of the intended construct (i.e. content validity).^11^ We did not predefine what would be considered a floor or ceiling effect but conventionally, significant floor and ceiling effects have been set at between 5% and 15%.^13^ The generally high scores in our study may accurately reflect participant satisfaction.

Most of the children in the study had eczema of mild to moderate disease, in keeping with the general population.^14^ However, 41.4% of participants did not meet the UK Working Party diagnostic criteria for atopic dermatitis, most participants were white and all participants were < 5 years of age, thus limiting the generalizability of the results.

This study is one of only five UK studies identified that have used questionnaires to assess participant emollient satisfaction. It is also the first UK study of its kind with child participants. We identified two international studies conducted in secondary care, a Japanese university hospital study,^15^ and a Korean university hospital outpatient study,^16^ both of which used questionnaires to characterize the views of adult patients with eczema surrounding emollients.

Previous studies have predominately compared two emollients without a rationale for questionnaire design, with each study using its own questionnaire. It is not possible to compare the validity of the ESQ with existing scales.

The ESQ appears to have good validity, suggesting that this questionnaire may be a useful tool with which to assess patients' emollient satisfaction. We recommend that future studies should score the ESQ in the same way as this study, on the basis that all items contribute equally to overall satisfaction. Future research should assess the reliability and validity of the ESQ in different populations and with other emollients. In particular, a content validity study is needed before the ESQ is recommended for wider use in research and clinical practice.

## Conclusion

Current evidence suggests that the ‘best’ emollient is the one that patients prefer after a period of testing.^4^ An emollient may be effective but only if acceptable and used by the patient. Our study suggests that, compared with Diprobase cream and Hydromol ointment, Aveeno lotion was preferred by parents. However, as only one of each emollient type were used in this trial, the findings may be specific to these particular emollients and not generalizable to a wider range of emollients.

**What's already known about this topic?**

- Emollients are the foundation of treatment in eczema but there are many types of emollient and there is a lack of head‐to‐head evidence to show one is any better than another.
- Robust patient self‐completed questionnaires can help assess emollient satisfaction, but none has published data on their validity.

**What does this study add?**

- We assess validity of the seven‐item ESQ, completed by parents for children with eczema who were aged < 5 years.
- Using data from the COMET trial, comparing Aveeno^®^ Lotion, Diprobase^®^ cream, Doublebase^®^ gel and Hydromol^®^ ointment, we present data supporting the validity of the ESQ in this patient population.

## Conflict of interest

MJR devised the Emollient Satisfaction Questionnaire. The other authors declare that they have no conflicts of interest.

## Funding

None.

## Ethics statement

Ethics approval was not applicable as data were from the previously approved COMET trial. Informed consent was not applicable.

## Data availability

Data are available from the corresponding author on reasonable request.

## Supporting information


**Data S1.** Intention for continued emollient use responses (Question 9), by study emollient.

**Figure S1.** Distribution of responses for emollient satisfaction questionnaire items 1–7 (*n* = 152).

**Figure S2.** Box and whisker plot to illustrate the relationship between overall emollient satisfaction score and total scaled emollient satisfaction score.

**Table S1.** Checklist for a reflective or formative questionnaire model.

**Table S2.** Descriptive statistics of individual emollient satisfaction questionnaire items 1–7.

**Table S3.** Total scaled emollient satisfaction scores, by study emollient (*n* = 139).

**Table S4.** Overall emollient satisfaction scores (Question 8), by study emollient.

**Table S5.** Intention for continued emollient use responses (Question 9), by study emollient.
