# Supplementary material for: Emollient satisfaction questionnaire: validation study in children with eczema
Source: Clin Exp Dermatol. 2022 May 16;47(7):1337–45. doi: 10.1111/ced.15189 (PMC9321994; doi:10.1111/ced.15189)
Supplement: Supplementary file 1 — Data S1. Intention for continued emollient use responses (Question 9), by study emollient. [file CED-47-1337-s001.docx]

COSMIN checklist: General recommendation for the design of a study on measurement properties

| ***Research aim*** | | ***Page*** |
| --- | --- | --- |
| 1 | Provide a clear research aim, including (1) the name and version of the PROM, (2) the target population, and (3) the measurement properties of interest | 3,4 |
| ***PROM*** | |  |
| 2 | Provide a clear description of the construct to be measured | 4 |
| 3 | Provide a clear description of the development process of the PROM, including a description of the target population for which the PROM was developed | 3,4,7 |
| 4 | The origin of the construct should be clear: provide theory, conceptual framework (i.e. reflective or formative model) or disease model used or clear rationale to define the construct to be measured | 4 |
| 5 | Provide a clear description of the structure of the PROM (i.e. the number of items and subscales included in the PROM, instructions given and response options) and its scoring algorithm | 4 |
| 6 | Provide a clear description of existing evidence on the quality of the PROM | 3,4 |
| 7 | Provide a clear description of the context of use* | 4 |
|  | ***Target population*** | |
| 8 | Provide a clear description of in- and exclusion criteria to select patients, e.g. in terms of disease condition and characteristics like age, gender, language or country, and setting (e.g. general population, primary care or hospital/rehabilitation care) | 3 |
| 9 | Provide a clear description of the method used to select the patients for the study (e.g. convenience, consecutive, or random) | 3 |
| 10 | Describe whether the selected sample is representing the target population in which the PROM will be used in terms of age, gender, important disease characteristics (e.g. severity, status, duration) | 7 |

* The context of use refers to the intended application of the PROM (e.g. for research or clinical practice), to a specific setting for which the PROM was developed (e.g. for use in a hospital or at home) or to a specific administration mode (e.g. paper or computer‐administered). If the PROM was developed for use across multiple contexts, this should be described.
