# Supplementary figures and images for: Emollient satisfaction questionnaire: validation study in children with eczema
Source: Clin Exp Dermatol. 2022 May 16;47(7):1337–45. doi: 10.1111/ced.15189 (PMC9321994; doi:10.1111/ced.15189)

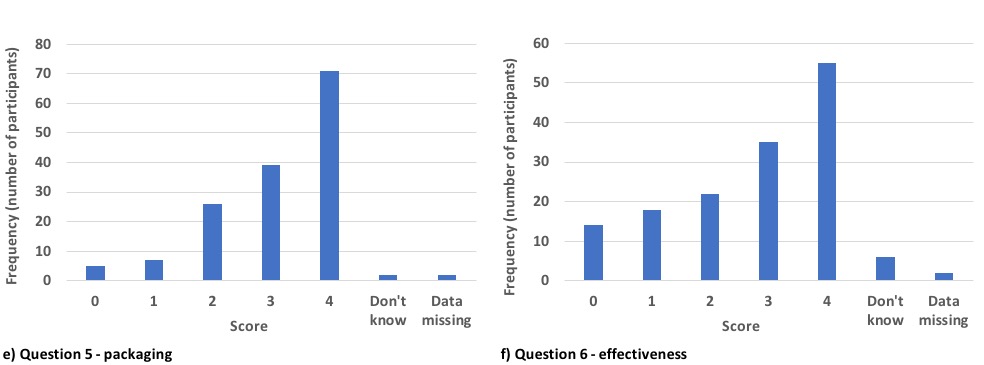

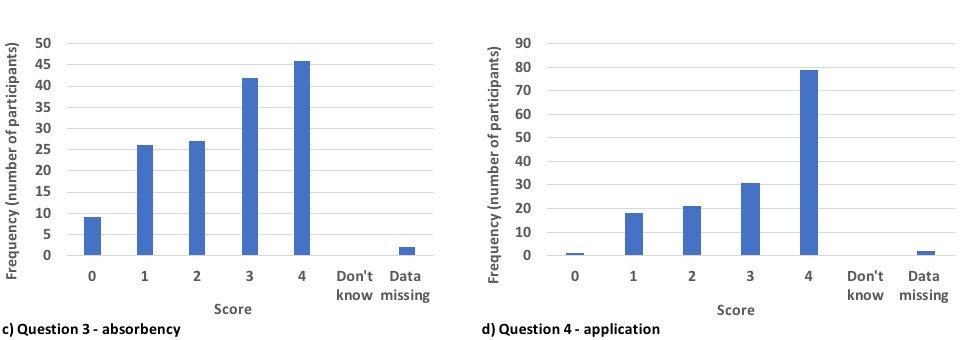

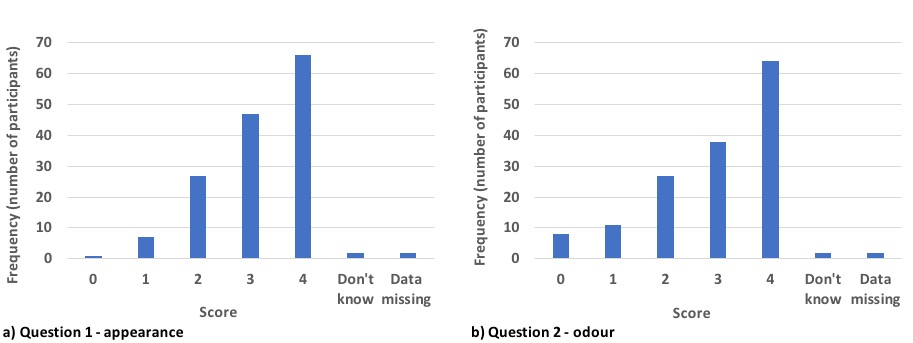
Figure S: Distribution of responses for emollient satisfaction questionnaire items 1-7 (n=152)


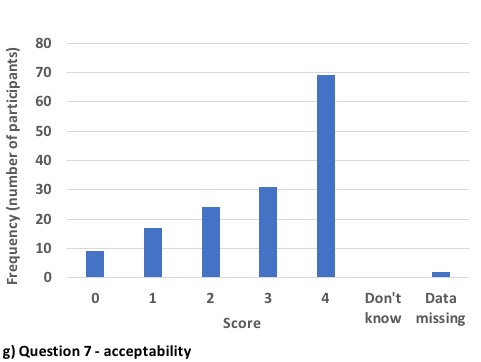

Supplement: Supplementary file 2 — Figure S1. Distribution of responses for emollient satisfaction questionnaire items 1–7 (n = 152). [file CED-47-1337-s008.docx]
