# Supplementary material for: Emollient satisfaction questionnaire: validation study in children with eczema
Source: Clin Exp Dermatol. 2022 May 16;47(7):1337–45. doi: 10.1111/ced.15189 (PMC9321994; doi:10.1111/ced.15189)

Figure S2: Box and whisker plot to illustrate the relationship between overall emollient satisfaction score and total scaled emollient satisfaction score.


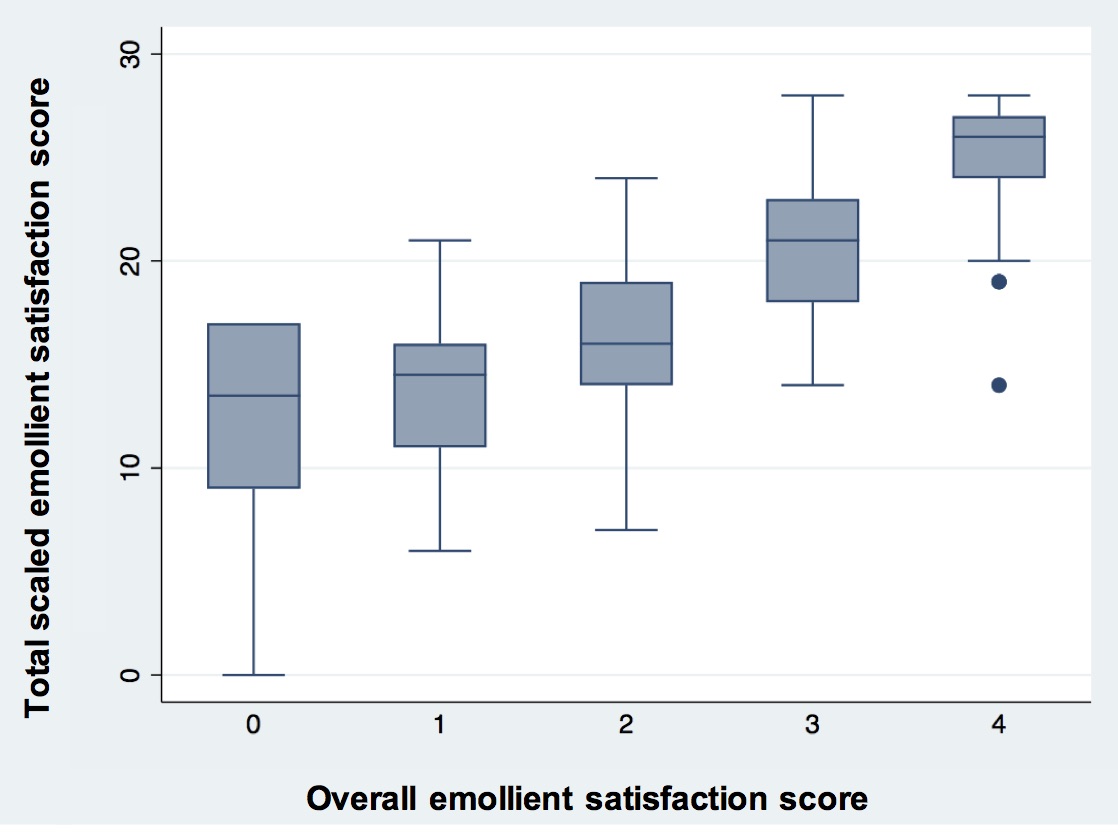

Supplement: Supplementary file 3 — Figure S2. Box and whisker plot to illustrate the relationship between overall emollient satisfaction score and total scaled emollient satisfaction score. [file CED-47-1337-s007.docx]
