# Supplementary material for: Emollient satisfaction questionnaire: validation study in children with eczema
Source: Clin Exp Dermatol. 2022 May 16;47(7):1337–45. doi: 10.1111/ced.15189 (PMC9321994; doi:10.1111/ced.15189)
Supplement: Supplementary file 4 — Table S1. Checklist for a reflective or formative questionnaire model. [file CED-47-1337-s002.docx]

Table S1: Checklist for a reflective or formative questionnaire model^11^

|  | **Checklist item** | **Application to “Emollient satisfaction”** |
| --- | --- | --- |
| 1 | Are the indicators (items) (A) defining characteristics or (B) manifestations of the construct?—“A” indicates a formative and “B” a reflective measurement model. | A: The definition, “the extent to which parents positively rated their given emollient for specific factors” indicates that the indicators (e.g. appearance, odour, absorbency) are defining characteristics of satisfaction with an emollient. |
| 2 | Would changes in the indicators/items cause changes in the construct or the other way around? | Satisfaction with an emollient cannot change independently of its indicators. Instead, the overall construct would change as a result of a change in one of the indicators, indicating a formative model. |
| 3 | Should each indicator capture exactly the same thing? | “No, but they share conceptual unity in terms of causing a common construct”, indicating formative. |
| 4 | Would dropping one of the indicators alter the conceptual domain of the construct? | “Yes”, indicating formative. If one of the indicators was removed, emollient satisfaction would be conceptually different from the construct with the indicator included. |
| 5 | Should a change in one of the indicators be associated with changes in the other indicators? “Yes” indicates reflective; “no” indicates formative. | “No”, indicating formative. The indicators of emollient satisfaction are potentially interrelated. For example, absorbency and ease of application may be related but not necessarily. Therefore, changes in one indicator can but do not need to be related to changes in the others. |
| 6 | Are the indicators expected to have the same antecedents and consequences? | “No”, indicating formative. |
