# Supplementary material for: Emollient satisfaction questionnaire: validation study in children with eczema
Source: Clin Exp Dermatol. 2022 May 16;47(7):1337–45. doi: 10.1111/ced.15189 (PMC9321994; doi:10.1111/ced.15189)
Supplement: Supplementary file 5 — Table S2. Descriptive statistics of individual emollient satisfaction questionnaire items 1–7. [file CED-47-1337-s005.docx]

| ESQ item | Mean (SD) score | Median (IQR) score |
| --- | --- | --- |
| 1. Appearance | 3.1 (0.9) | 3.0 (3-4) |
| 2. Odour | 2.9 (1.2) | 3.0 (2-4) |
| 3. Absorbency | 2.6 (1.3) | 3.0 (2-4) |
| 4. Application | 3.1 (1.1) | 4.0 (2-4) |
| 5. Packaging | 3.1 (1.1) | 3.0 (2-4) |
| 6. Effectiveness | 2.7 (1.4) | 3.0 (2-4) |
| 7. Acceptability | 2.9 (1.3) | 3.0 (2-4) |

Table S2: Descriptive statistics of individual emollient satisfaction questionnaire items 1-7.
