# Supplementary material for: Emollient satisfaction questionnaire: validation study in children with eczema
Source: Clin Exp Dermatol. 2022 May 16;47(7):1337–45. doi: 10.1111/ced.15189 (PMC9321994; doi:10.1111/ced.15189)
Supplement: Supplementary file 6 — Table S3. Total scaled emollient satisfaction scores, by study emollient (n = 139). [file CED-47-1337-s006.docx]

Table S3: Total scaled emollient satisfaction scores, by study emollient (n=139).

| **Study emollient** | **Number in group** | **Mean (SD) total scaled emollient satisfaction score** | **Median (IQR) total scaled emollient satisfaction score** |
| --- | --- | --- | --- |
| Aveeno lotion | 33 | 23.5 (3.9) | 25.0 (21-27) |
| Diprobase cream | 38 | 19.4 (6.2) | 21.0 (14.75-24) |
| Doublebase gel | 37 | 21.1 (6.5) | 23.0 (16.5-27) |
| Hydromol ointment | 31 | 18.4 (4.6) | 18.0 (14-22) |
| **All** | 139 | 20.6 (5.7) | 22.0 (16-26) |
