# Supplementary material for: Emollient satisfaction questionnaire: validation study in children with eczema
Source: Clin Exp Dermatol. 2022 May 16;47(7):1337–45. doi: 10.1111/ced.15189 (PMC9321994; doi:10.1111/ced.15189)
Supplement: Supplementary file 7 — Table S4. Overall emollient satisfaction scores (Question 8), by study emollient. [file CED-47-1337-s004.docx]

|  | **Number (row %) of participants** | | | | | | |
| --- | --- | --- | --- | --- | --- | --- | --- |
|  | Overall emollient satisfaction score | | | | | | |
| **Allocated emollient** | Very dissatisfied | Dissatisfied | Neither satisfied nor dissatisfied | *Mostly* satisfied | Very satisfied | Data missing | Total |
| Aveeno lotion | 0 (0.0) | 1 (2.4) | 8 (19.5) | 8 (19.5) | 23 (56.1) | *1 (2.4)* | 41 |
| Diprobase cream | 3 (7.7) | 10 (25.6) | 5 (12.8) | 9 (23.1) | 12 (30.8) | *0 (0.0)* | 39 |
| Doublebase gel | 3 (7.7) | 6 (15.4) | 5 (12.8) | 10 (25.6) | 15 (38.5) | *0 (0.0)* | 39 |
| Hydromol ointment | 2 (6.1) | 3 (9.1) | 5 (15.2) | 13 (39.4) | 10 (30.3) | *0 (0.0)* | 33 |
| **Total** | 60 (39.5) | 40 (26.3) | 23 (15.1) | 20 (13.2) | 8 (5.3) | 1 (0.7) | 152 |

Table S4: Overall emollient satisfaction scores (question 8), by study emollient.
