# Supplementary material for: Emollient satisfaction questionnaire: validation study in children with eczema
Source: Clin Exp Dermatol. 2022 May 16;47(7):1337–45. doi: 10.1111/ced.15189 (PMC9321994; doi:10.1111/ced.15189)
Supplement: Supplementary file 8 — Table S5. Intention for continued emollient use responses (Question 9), by study emollient. [file CED-47-1337-s003.docx]

Table S5: Intention for continued emollient use responses (question 9), by study emollient.

|  | **Number (row %) of participants** | | | | |
| --- | --- | --- | --- | --- | --- |
|  | Intention for continued emollient use | | | | |
| **Allocated emollient** | Yes | No | Not Sure | *Data missing* | Total |
| Aveeno lotion | 27 (65.9) | 3 (7.3) | 9 (22.0) | *2 (4.9)* | 41 |
| Diprobase cream | 19 (48.7) | 15 (38.5) | 5 (12.8) | *0 (0.0)* | 39 |
| Doublebase gel | 24 (61.5) | 9 (23.1) | 6 (15.4) | *0 (0.0)* | 39 |
| Hydromol ointment | 22 (66.7) | 5 (15.2) | 6 (18.2) | *0 (0.0)* | 33 |
| **Total** | 92 (60.5) | 32 (21.1) | 26 (17.1) | 2 (1.3) | 152 |
